# *Harmonia sedecimnotata* (F.): Predatory potential, biology, life table, molecular characterization, and field evaluation against *Aphis gossypii* Glover

**DOI:** 10.1038/s41598-020-59809-3

**Published:** 2020-02-20

**Authors:** T. Boopathi, S. B. Singh, S. K. Dutta, V. Dayal, A. R. Singh, S. Chowdhury, Y. Ramakrishna, R. Aravintharaj, I. Shakuntala, K. Lalhruaipuii

**Affiliations:** 1 ICAR Research Complex for NEH Region, Mizoram Centre, Kolasib, 796081 Mizoram India; 2 Central Agricultural University, Imphal, 795004 Manipur India; 3 ICAR Research Complex for NEH Region, Umroi Road, Umiam, 793103 Meghalaya India; 4 ICAR Research Complex for NEH Region, Sikkim Centre, Tadong, Gangtok, 737102 Sikkim India; 5 ICAR-Krishi Vigyan Kendra, ICAR Research Complex for NEH Region, Ukhrul, 795145 Manipur India; 6 Department of Plant Biotechnology and Molecular Biology, Tamil Nadu Agricultural University, Coimbatore, 641003 Tamil Nadu India

**Keywords:** Sequencing, Entomology

## Abstract

The ladybird beetle, *Harmonia sedecimnotata* (F.) was studied in biology, life table, consumption rates, molecular characterization, and field evaluation. The net reproductive rate (*R*_0_), based on the age-stage and two-sex life table, was 43.2 eggs/individual. The female adults lived longer (68.1 d) than the male adults (62.9 d). The rate of consumption increased with progress in each stage of development. Compared to the other larval stages of the predator, the fourth stadium consumed most quantities of *Aphis gossypii* Glover nymphs (Hemiptera: Aphididae) (200.4). Both female (2214.6) and male (1792.4) consumed more prey (nymphs) than larvae. The net rate of consumption was 1458.92 nymphs of melon aphids. There was no variation in the sequences of the two nucleotides out of 583 bp, *H sedecimnotata* China (EU392410) and India (MG720024). Our investigations demonstrated that inoculative release of 30 or 40 or 50 adults per 100 m^2^ attained high reduction of aphids (>90%). Thus, it may be recommended the release rate of 40 adults per 100 m^2^ to suppress the eggplant aphid population. *H. sedecimnotata* is therefore one of the most promising biological control agents for cotton aphids that can be achieved for instant control through an inoculative release of adults.

## Introduction

Many of the sucking insect pests cause severe damage to the crop by injecting toxins through their salivary secretions^1–3^ and serve as vectors for many plant diseases as well. In both field and protected conditions, they are economically important insect pests^1,4^. The aphid is one of the most important sucking pests and in many ways lower yields of agricultural and horticultural crops. High population densities may damage plants by removing plant nutrients to cause withering and death. Aphids inject toxins from their salivary secretion that cause curling, crinkling and malformations of the plant. They cause secondary plant injury by transmitting many plant diseases. Farmers rely on a variety of insecticides to control these pests^5^. Most of them adversely affect natural enemies such as predators, parasitoids, entomopathogens and have also resulted in residue, resurgence, resistance, and unwanted effects on human and non-target organisms^6,7^. Biological control is a good substitute for toxic insecticides that can protect plants, human beings and the environment^8–11^. Among the most important beneficial insects that feed on sucking insect pests are coccinellid predators^12^. There are several habitats where the coccinellid beetles are found^13^. They feed on sap-sucking insects like aphids, mealybugs, scales, thrips and mites^14^, as well as other soft-body insects^15^.

More than 5,200 species have been described worldwide in the Coccinellidae family^14^. Around 4,000 predatory ladybird beetle species have been reported from the Indo-Pak subcontinent, including >300 species^16^. The harlequin lady beetle, *Harmonia axyridis* Pallas (Coleoptera: Coccinellidae) in the genus *Harmonia* Mulsant is one of the most popular biocontrol agents for greenhouse pest that has become a dangerous invader^17^. Very few studies have been carried out in the other species of the *Harmonia* genus. The sporadic occurrence of *Harmonia sedecimnotata* (F.) (Coleoptera: Coccinellidae) from India has recently been reported^18^. Most of the *H. sedecimnotata* occurs in okra, eggplant, chili, tomato, guava, ornamental, etc.^18^. Polyphagous ladybird predators who feed on various aphid prey species on crop plants exhibit biological fitness variations and are therefore likely to vary in their genotypes^19,20^. An important source for biocontrol agents will be the most effective genotypes of a predator species with higher predation potential^21^. In comparison to other insect species, limited molecular studies were carried out in the members of the Coccinellinae subfamily of the Coccinellidae^20,22^. A partial COI gene was identified and the taxonomic relationship of 16 species was analyzed through four subfamilies^23^. The 5′ region of the COI gene is considered informative and has also been commonly used to detect molecular genetic variation within and between species^24^. For assessing the host-associated variations for insect species, the COI gene has also been effectively employed^3,25^. In this investigation, we used the COI gene for molecular characterization of *H. sedecimnotata*. The purpose of this study is to select aspects of the consumption rate, biology, life table studies of the *H. sedecimnotata* fed on *Aphis gossypii* Glover (Hemiptera: Aphididae), field evaluation and its molecular characterization. Hopefully, this study would help to enhance Integrated Pest Management (IPM) strategies by using this predator as a potential biological control agent for aphid management.

## Results

### Consumption rate

**Figure 1:**
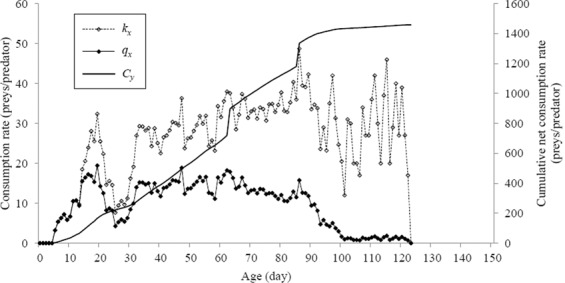
The *k*_*x*_ (age-specific consumption rate), *q*_*x*_ (the age-specific net consumption rate) and *C*_*y*_ (the cumulative net consumption rate) of *Harmonia sedecimnotata* fed on *Aphis gossypii*. The highest values of *k*_*x*_ and *q*_*x*_ occur at age 86 d and 19 d, respectively.

The various larval stages, as well as the adult stage, had significant effects on per-day, percentage and total consumption of *A. gossypii*. The age-specific consumption rate, *k*_*x*_ (mean number of aphids fed by *H. sedecimnotata* age *x*) and age-specific net consumption rate, *q*_*x*_ (weighted number of prey fed by *H. sedecimnotata* age *x*) were formulated by considering sex and stage differentiation (Fig. 1). The net consumption rate (*C*_0_), the transformation rate (*Q*_*p*_) and the total consumption of the cohort were 1458.92, 33.77 and 36473 nymphs of melon aphids respectively (Table 1).

**Table 1:** Consumption rate (average ± standard error) of *Harmonia sedecimnotata* reared on *Aphis gossypii*.

Stage and statistics		Consumption rate (No. of aphids consumed per individual)
***Stages of Harmonia sedecimnotata***		
Larval stage	1st stadium	23.84d ± 1.24
	2nd stadium	25.76d ± 3.22
	3rd stadium	69.57c ± 6.48
	4th stadium	237.54b ± 19.72
Pre-adult stage	1st to 4th stadia	230.88 ± 30.98
Adult stage	Male	249.68b ± 65.12
	Female	978.36a ± 165.61
Total lifespan of an individual	Male	6242 ± 207.74
	Female	24459 ± 239.94
***Statistics of consumption rate***		
Total consumption of the cohort		36473
Net predation rate (*C*_*o*_)		1458.92
Transformation rate (*Q*_*p*_)		33.77

Data in interaction analyzed with least squares means (LSD) and means separated with a standard error of the mean at *P* ≤ 0.01. In a column means followed by the same letter are not significantly different at *P* ≤ 0.001 as determined by Tukey’s HSD test.

**Figure 2:**
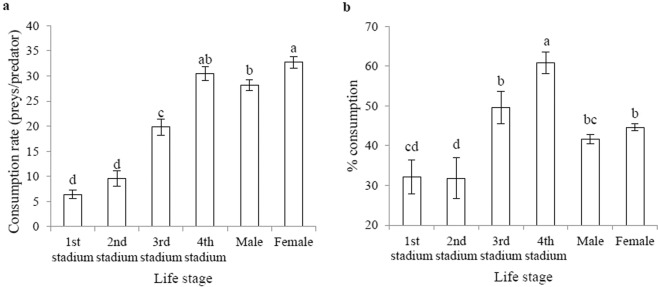
(**a**) Per-day consumption (average ± standard error) and (**b**) percent consumption (average ± standard error) of prey (*Aphis gossypii*) by *Harmonia sedecimnotata*. Data in interaction analyzed with least squares means (LSD) and means separated with a standard error of the mean at *P* < 0.01. Each bar bearing different letters are not significantly different as determined by Tukey’s HSD test. Analysis of variance (ANOVA) for per-day consumption: *F* = 75.519, df = 5, 54, *P* < 0.001.

The rate of consumption per day was significantly different (*F* = 75.519; df = 5, 54; *P* < 0.001) (Fig. 2a). In the fourth stadium it was found highest compared to other stadia. The daily consumption rate among adult beetles was the highest in female adults compared to male adults. Percent consumption was significantly varied (Fig. 2b). Fourth stadium percent consumption was found to be higher than three other stadia. The second stadium was the least percentage of aphids consumption. In adult beetles, aphid intake was found to be the highest in female adults compared with male adults.

**Figure 3:**
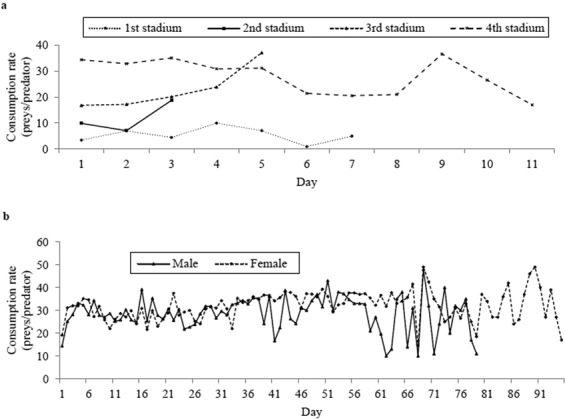
Daily consumption of prey (*Aphis gossypii*) by (**a**) larvae and (**b**) adult beetles of *Harmonia sedecimnotata*.

During the 4th day and the 6th day the first stadium consumed more and least prey respectively (Fig. 3a). At 3rd day the second stadium killed more prey and at 2nd day the lowest. During the 5th day the third stadium consumed most aphids and during the 1st day the least. The fourth stadium preyed most aphids at 9th day and less at 11th day. Male adult fed more aphids on the 69th day and less on 62nd and 68th day (Fig. 3b). Female adult ate most aphids at 69th and 90th day and it was least at 68th day. The most daily consumption rate in adults was higher than in grubs.

**Figure 4:**
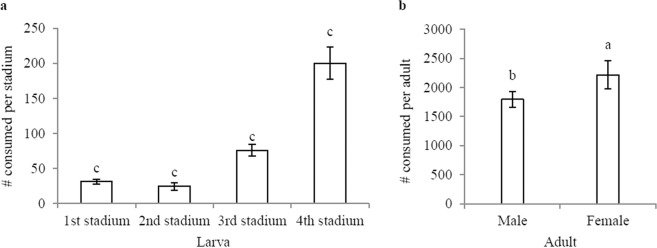
Total consumption (±standard error) of prey (*Aphis gossypii*) by both larvae (**a**) and adults (**b**) of *Harmonia sedecimnotata*. Data in interaction analyzed with least squares means (LSD) and means separated with standard error of the mean at *P* < 0.01. Each bar bearing different letters are not significantly different as determined by Tukey’s HSD test. ANOVA: *F* = 77.429, df = 5, 54, *P* < 0.001.

The total consumption rate (*F* = 77.429; df = 5, 54; *P* < 0.001) was significantly different (Fig. 4). During the first to fourth stadium, a drastic increase in the total consumption rate was found (Fig. 4a). The total number of aphids consumed per larva (±SE) during the larval stage was 230.88 ± 30.98 (Table 1). Compared to the second stadium, the 1st stadium killed more aphids but less than the 3rd and 4th stadium (Fig. 4a). The 2nd stadium had fewer aphids than the 1st, 3rd, and 4th stadia. The 3rd stadium consumed more aphids than 2nd and 1st stadia, but less than the 4th stadium. The 4th stadium killed more prey than any of the preceding stages. Most aphids were consumed by female adults compared to male adults (Fig. 4b). Because they lived longer, both females and males killed more prey than larvae.

### Life tables and population parameters

All 85 *H. sedecimnotata* eggs hatched. Table 2 presents an average developmental period for egg, larva, pupa, longevity of adults and fecundity. It was observed that the predator lay eggs in groups and the duration was around 5.0 d. The average overall development period (±SE) was 29.6 ± 0.62 d. The average number of eggs laid per female was 75.7.

**Table 2:** Developmental period, oviposition period, adult longevities and fecundity (average ± standard error) of *Harmonia sedecimnotata* fed on *Aphis gossypii*.

Stages and statistics	n	Average ± standard error
***Developmental period (day)***		
Egg	85	5.0 ± 0.00
1st stadium	85	5.7 ± 0.30
2nd stadium	81	2.6 ± 0.22
3rd stadium	78	3.9 ± 0.28
4th stadium	73	7.3 ± 0.75
Larva (1st to 4th stadia)	73	19.5 ± 1.01
Pupa	69	5.1 ± 0.59
Total development period	69	29.6 ± 0.62
***Adult longevity and oviposition periods (day)***		
Female	41	68.1 ± 6.56
Male	25	62.9 ± 0.62
Pre-oviposition	41	24.7 ± 6.18
Oviposition	41	36.7 ± 3.00
Post-oviposition	41	6.7 ± 5.32
*Statistics*		
Fecundity (eggs/individual)		75.7 ± 4.99
Daily maximum fecundity		52
Lifelong maximum fecundity		1080

Data in interaction analyzed with least squares means (LSD) and means separated with a standard error of the mean at *P* ≤ 0.01.

**Figure 5:**
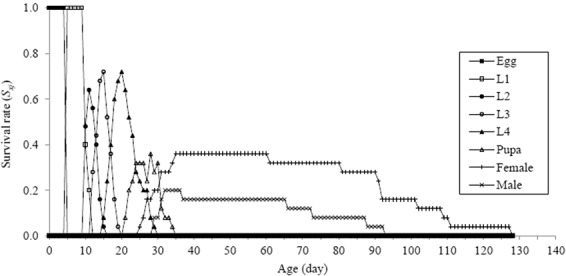
The age-stage specific survival rates of *Harmonia sedecimnotata* fed on *Aphis gossypii*. The first adult began at 25 d and the survival rate was 0.36.

**Figure 6:**
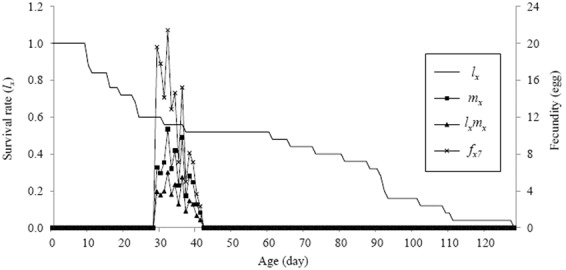
The *l*_*x*_ (the age-specific survival rates), *m*_*x*_ (the age-specific fecundities), *l*_*x*_*m*_*x*_ (the age-specific maternities) and *f*_*x7*_ (the age-stage specific fecundities) of *Harmonia sedecimnotata* fed on *Aphis gossypii*.

The probability of age-stage survival rate (*S*_*xj*_) for eggs was 1.0. The probability of *S*_*xj*_ was differed between larval stadia and also differed between adults. The overlap between different stages showed that more variability was found among individuals in the developmental rates (Fig. 5). The age-specific fecundity (*m*_*x*_), age - specific survival (*l*_*x*_), and age - specific maternity (*l*_*x*_*m*_*x*_) ranged from 0.04 to 0.88, 1.62 to 10.71 and 0.84 to 6.00, respectively (Fig. 6). The offspring of an individual *H. sedecimnotata* ranged from 2.33 to 21.43. As the only female lays eggs, a single *f*_*x7*_ curve was noticed. The estimated values for the intrinsic rate of increase (*r*), the finite rate of increase (*λ*), the net rate of reproduction (*R*_0_) and the mean time of generation (*T*) for all individuals were 0.1096 day, 1.1159 day, 43.20 eggs/individual and 34.35 day, respectively.

### Molecular characterization

**Figure 7:**
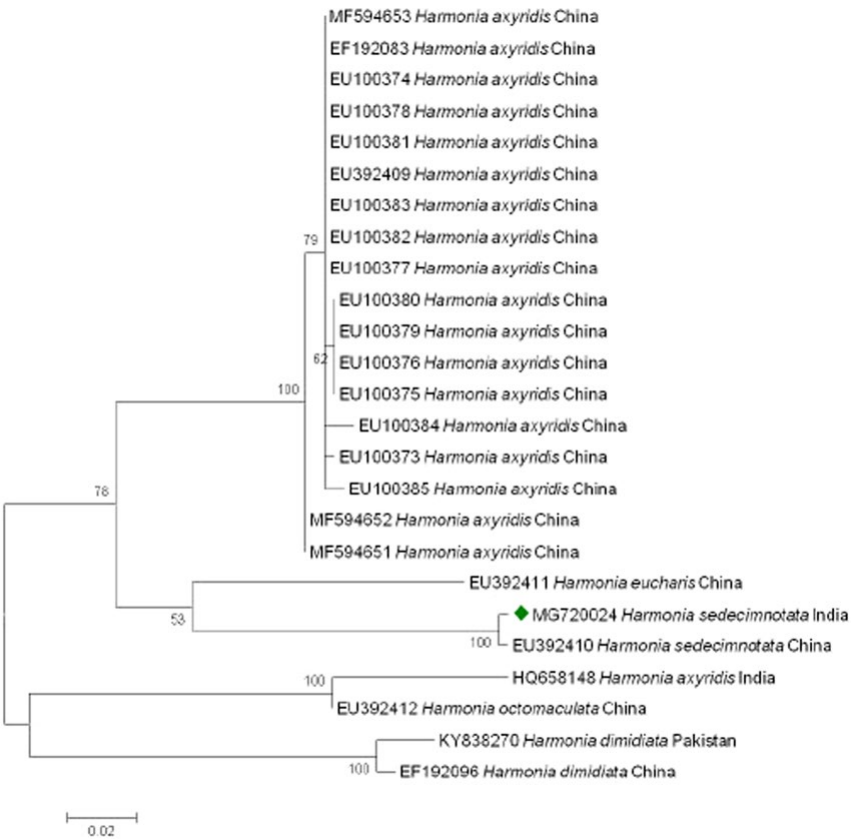
Phylogenetic tree inferred from the Neighbor-Joining (NJ) analysis based on the COI dataset. Bootstrap values are shown near the nodes of individual branches. Only bootstrap values larger than 60% are shown. The sequences in diamond was generated in this study and deposited in the NCBI database.

To conclude the phylogenetic relationship between *H. sedecimnotata* and other species of *Harmonia*, the COX gene region was analyzed. When alignment gaps were added, 583 bases were analyzed. The Neighbor-Joining (NJ) trees contributed low as well as high bootstrap values of *COX* gene region. Alignment of the sequences *H. sedecimnotata* China (EU392410) and India (MG720024) shows that there was no variation in two nucleotides outs of 583 bp (Fig. 7).

### Field evaluation of *Harmonia sedecimnotata* against *Aphis gossypii* on eggplant

The aphid populations increased in the control during two seasons (34.10 ± 0.94 and 42.04 ± 0.78/leaf, respectively) on the eggplant (Table 3). During two seasons, a gradual decrease in aphid infestation was observed in all treatments. A percentage reduction in an aphid population in both seasons influenced by release rates. The aphid infestation in both seasons decreased at the release rate of 40 and 50 adults. After 2 releases of 30 or 40 or 50 adults in both seasons, percentage reduction in an aphid population reached >90%. After second release in release rate 40 and 50 adults were the highest percentage reduction in the aphid population in both season 1 and 2.

**Table 3:** Field efficacy of *Harmonia sedecimnotata* against *Aphis gossypii* population on eggplant during two seasons.

Release rate per 100 m^2^	Pretreatment count (No. of aphid/leaf ± SE)^$^	I release^#^ (average ± standard error)		II release^#^ (average ± standard error)	
		No. of aphid/leaf ^$^	% reduction over control^@^	No. of aphid/leaf ^$^	% reduction over control^@^
***Season 1***					
10	13.32 ± 1.18 (3.65)a	9.38 ± 0.61 (3.06)d	53.11 ± 3.86 (46.79)d	6.64 ± 0.59 (2.58)d	79.15 ± 1.57 (62.83)d
20	14.52 ± 0.90 (3.81)a	8.50 ± 0.49 (2.92)cd	58.23 ± 2.32 (49.73)cd	3.44 ± 0.19 (1.85)c	88.44 ± 0.97 (70.12)c
30	15.14 ± 0.52 (3.89)a	7.22 ± 0.16 (2.69)c	64.55 ± 1.66 (53.46)c	2.40 ± 0.22 (1.55)b	91.95 ± 1.02 (73.52)b
40	16.62 ± 0.78 (4.08)a	5.34 ± 0.37 (2.31)b	73.65 ± 1.90 (59.11)b	0.96 ± 0.04 (0.98)a	96.90 ± 0.12 (79.86)a
50	14.56 ± 0.40 (3.82)a	3.74 ± 0.28 (1.93)a	81.75 ± 1.62 (64.71)a	0.56 ± 0.05 (0.75)a	98.20 ± 0.24 (82.30)a
Control	13.38 ± 0.48 (3.66)a	21.14 ± 0.65 (4.60)e	—	34.10 ± 0.94 (5.84)e	—
df	5,20	5,20	4,16	5,20	4,16
*F* value	1.65	87.09	20.17	434.81	62.38
*P* value	0.194 ns	<0.001	<0.001	<0.001	<0.001
***Season 2***					
10	20.92 ± 0.14 (4.57)bc	12.66 ± 0.58 (3.56)c	56.84 ±1.45 (48.93)c	9.16 ± 0.57 (3.03)e	77.77 ± 1.29 (61.87)e
20	22.54 ± 0.46 (4.75)d	11.78 ± 0.31 (3.43)c	59.26 ± 1.53 (50.33)c	5.50 ± 0.12 (2.35)d	85.88 ± 0.59 (67.93)d
30	18.52 ± 0.33 (4.30)a	11.24 ± 0.36 (3.35)c	61.05 ± 1.50 (51.38)c	2.90 ± 0.21 (1.70)c	92.45 ± 0.68 (74.05)c
40	21.56 ± 0.62 (4.64)cd	8.62 ± 0.44 (2.94)b	70.50 ± 1.36 (57.10)b	1.00 ± 0.12 (1.00)b	97.54 ± 0.26 (80.97)b
50	20.94 ± 0.29 (4.58)bc	5.50 ± 0.46 (2.35)a	80.42 ± 2.08 (63.74)a	0.54 ± 0.11 (0.73)a	98.63 ± 0.29 (83.29)a
Control	19.86 ± 0.45 (4.46)ab	30.40d ± 0.78 (5.51)d	—	42.04e ± 0.78 (6.48)f	—
df	5,20	5,20	4,16	5,20	4,16
*F* value	7.17	118.13	25.07	585.62	116.47
*P* value	<0.001	<0.001	<0.001	<0.001	<0.001

ns–non-significant, ^#^mean of five replications, ^$,@^ figures in parentheses are square root and arc sine transformed values, respectively. In parentheses, the transformed values followed by the same letter are not significantly different at *P* ≤ 0.001 as determined by Tukey’s HSD test.

## Discussion

By feeding on *A. gossypii* alone, the coccinellid predator (*H. sedecimnotata*) could reproduce. Both larvae and adults were voracious feeders on all stages of cotton aphids. The prey consumption values were significantly higher with the advancement in each larval stage except the second stadium, which consumed fewer aphids. An increase in feeding potential with age of the larvae was demonstrated in *Cryptolaemus montrouzieri* Mulsant (Coleoptera: Coccinellidae)^26^ which is in accordance with our findings. Compared to the other larval stages of the predator, the fourth stadium consumed most aphids. These results are consistent with earlier findings that reported more than 653.25 *Cervaphis quercus* (Takahashi) (Hemiptera: Aphididae)^27^, 88.75 *Brevicoryne brassicae* (L.) (Hemiptera: Aphididae)^28^ and 92.13 *Tuberculatus nervatus* Chakrabarti and Raychaudhuri (Hemiptera: Aphididae)^29^ were consumed in the fourth stadium of *Harmonia dimidiata* (F.) (Coleoptera: Coccinellidae). In the later larval stadia, the consumption rate increased and continued in the adult stage, resulting in one individual being able to kill 4338.6 aphids throughout life.

Most aphids were consumed by the females (*F* = 77.429; df = 5, 54; *P* < 0.001) compared to male and larval stages. This is in line with the earlier finding that *Cheilomenes sexmaculata* (F.) (Coleoptera: Coccinellidae) female killed a maximum of 1624, *Aphis craccivora* Koch (Hemiptera: Aphididae) than larval stages and male^30^. The consumption rate of adults in the current investigation was higher than the larval stages. Similarly, when fed on *A. craccivora*, the feeding effectiveness of *C. sexmaculata* adults was higher than that of larvae^30^. Using the life tables due to non-predatory stages (eggs and pupae), two gaps in consumption rate were depicted. This will determine the life tables’ significance and also helped to determine the release time in a biocontrol program.

An overall adult consumption rate was higher than the larva feeding rate because adults were able to live longer in the ecosystems and search for their prey. In addition, more eggs were laid by the female beetles and emerged larvae could search for their prey. *C. sexmaculata* adults had a higher consumption rate than larvae fed on *A. craccivora*^30^. The release of coccinellid adults (@ 10 adults/tree or 1500 adults/ha) could suppress the populations of sucking pests^31^. Thus, adults have the potential to be used to suppress sucking pests in biocontrol programs.

The female adults lived longer than the male adults in our investigation. A longer female longevity (81.0 d) and male longevity (73.0 d) of *H. dimidiata* was demonstrated when reared on *B. brassicae*^28^ than our results indicate shorter lifespans fed on *A. gossypii* by *H. sedecimnotata*. Our study showed that *H. sedecimnotata* could reproduce a lifelong fecundity of 1080 eggs. The age-specific fecundity (*m*_*x*_) and age-specific maternity (*l*_*x*_*m*_*x*_) showed irregular peaks in female fecundity similar to those shown by *Lemnia biplagiata* (Swartz) (Coleoptera: Coccinellidae), another coccinellid^32^. The first emerged adult’s survival rate (*l*_*a*_) was 0.65. Since its most important hypothesis, this will help in further statistical analysis.

The development rate among individuals and the role of male individuals in a population is ignored by traditional female age-specific life tables^21^. The stage survival rates and overlaps would not have been found if traditional life tables were used in this study. The life table of *Tamarixia radiata* Waterson (Hymenoptera: Eulophidae) was recorded using the female age-specific life table^33^. Since the female age-specific life table avoids the male population and could not adequately explain the stage difference, it is possible to obtain ‘adult age’ in measuring population parameters and no clear *F* and *R*_0_ relationship. Problems were addressed in adapting female life tables to insect populations^34^. It was possible to observe the stage differentiation of *H. sedecimnotata* by using the age-stage, two-sex life table and the emergence and termination of various stages. Nevertheless, this gives a single age-specific survival rate when the survival rates from different stages are combined. At an older age, the survival rate can only be equal or lower than at an earlier age (*x*). It is also possible to observe certain monotonous properties of the *l*_*x*_ curve in all studies focused on age-specific life table.

Different methods and parameters were used to estimate population growth potential and insect mass rearing efficiencies. For example, *Adalia bipunctata* L. (Coleoptera: Coccinellidae) can be grown massively on *Encallipterus tillae* (L.) and *Euceraphis betulae* (L.) (Hemiptera: Aphididae) as they were more suitable prey for *A. bipunctata* based on their rates of development and survival and more abundance in ecosystems^35^. Based on data from the life table, *Scymnus apetzi* Mulsant (Coleoptera: Coccinellidae), *S. subvillosus* (Goeze), and *Exochomus nigromaculatus* (Goeze) (Coleoptera: Coccinellidae) have the potential to be used in the IPM program to manage *Hyalopterus pruni* (Geoffer) (Hemiptera: Aphididae)^36^.

The mitochondrial genes and the 16 S rDNA gene were used to investigate the insect relationships between the genus level and the level of the species^37^. The mitochondrial genes provide support throughout the topology below the genus level and this will help confirm the species’ current positions in coccinellid evolution. In this investigation, 100 percent similarity was found between the sequences of the two nucleotides, *H. sedecimnotata* China (EU392410) and India (MG720024) out of 583 bp. These patterns might be controlled by the function of the gene and might also be caused by mitochondrial genes^38^. The 16 S rDNA was used to investigate higher taxonomic levels of *Anisoptera* and Odonata groups^39^. Most important to know about the entire phylogeny of ladybird beetles is the incorporation of morphological characteristics, ecological characteristics and DNA sequence.

*Harmonia sedecimnotata* is suitable for mass rear in the laboratory, which is an essential requirement for inoculative releases. The most important considerations for inoculation are the number of beetles to be released and the timing of releases. Inoculative releases of *H. sedecimnotata* showed a higher reduction in the aphid population in the eggplant. Earlier, *Rodolia iceryae* Janson (Coleoptera: Coccinellidae) was reintroduced as an inoculative release into a plantation where the beetle was absent, and within one year the population of *lcerya pattersoni* Newstead (Hemiptera: Monophlebidae) was reduced to below the economic threshold^40^. Ladybird beetle predation can slow population growth and reduce aphid densities in the early growing season and the growth of a crop^41^. After 3 releases of 30 *Hippodamia varigata* (Goeze) (Coleoptera: Coccinellidae) larvae on rose leaves and flower buds, more than 95% aphid reduction was achieved^42^. In this study, the release rates of 30 or 40 or 50 adults on eggplant achieved a high reduction in aphid (>90%). Thus, to suppress the aphid population on eggplant, we recommend the release rate of 40 adults per 100 m^2^.

The life tables and consumption rates were the most important parameters for predator-prey dynamics models, but most pest management based biocontrol programs do not use these life tables. We determined the development rate, survival rate, life tables, and biology of *H. sedecimnotata* fed on *A. gossypii* in our investigations, and this predator also had higher consumption rates. In order to know the complete phylogeny of the ladybird beetles, research on ecological character, morphological character and character in the DNA sequence is essential. *H. sedecimnotata* is a good biocontrol agent that can be used in an inoculative release (40 beetles/100 m^2^) to control *A. gossypii*. This biological information revealed that *H. sedecimnotata* has the potential to be exploited on various crops in India to control sucking insects.

## Methods

### *Aphis gossypii* source

An *A. gossypii* culture was maintained at a temperature of 25 ± 5 °C and 75 ± 5% RH on potted okra plants in a polyhouse of 20 mesh cm^−2^. In the experimental field of the ICAR Research Complex for NEH Region, Mizoram Centre, Kolasib, Mizoram, India, Okra (cv. Parbhani Kranti) was grown on a plot sized 10 × 10 m. Okra plants were exposed to natural aphids infestation and on okra plants the aphids began colonizing.

### *Harmonia sedecimnotata* source

Adult beetles were obtained from various host plants and grown on aphids at 23 ± 5 °C temperature and 70 ± 5% RH in the Biological Control Laboratory, ICAR Research Complex for the NEH Region, Mizoram Centre, Kolasib, Mizoram, India. The beetles were fed daily with aphids in plastic containers (7 cm in diameter and 10 cm height) covered by a fine nylon netting for ventilation.

### Studies of the consumption rate, biology and life tables

The 10 pairs of males and females that emerged freshly were kept in a plastic container. The plastic containers were secured for ventilation by fine nylon netting and kept at a temperature of 23 ± 1 °C at 70 ± 1% RH. These pairs fed nymphs of melon aphids along with okra leave as food for the nymphs. For the biology and life tables, 85 freshly laid eggs were collected and held in a plastic container under the same conditions. Newly emerging larvae were kept for individual rearing in a new plastic container. The first stadium, second stadium, third stadium, fourth stadium, and adults were provided with 30, 40, 50, 75, and 100 nymphs of melon aphids per cup daily. The observations on prey consumption were recorded daily in all larval stages until the stadium changes and in the case of adult beetles until death. Small plastic pipette tubes were kept for oviposition (1.2 cm in diameter, 3 cm in length). The time taken from egg laying to hatching was recorded as well as the duration of larval and pupal stages. Also calculated were the total life cycle, pre-oviposition, oviposition, post-oviposition, fecundity, and adult longevity. The experiment was repeated twice (November to February 2013 and 2014) under laboratory conditions and the mean of both experiments was used to determine life table and consumption rate of *H. sedecimnotata*.

### Analysis of life tables

Based on raw data, the age-stage and two-sex life tables^43,44^ were shown using the TWOSEX-MSChart computer program^45^. It calculated the age-stage-specific survival rate (*S*_*xj*_), the age-stage-specific fecundity (*f*_*xj*_), the age-specific survival rate (*l*_*x*_), the age-specific fecundity (*m*_*x*_), the net reproductive rate (*R*_0_), the intrinsic rate of increase (*r*), the finite rate of increase (*λ*), and the mean generation time (*T*)^43^.

### Analysis of consumption rate

Total, per-day and percentage of aphid consumption was calculated for each stage of life of predatory beetle. The daily consumption rates were calculated using the CONSUME-MSChart computer program^46^. It calculated the age-specific consumption rate (*k*_*x*_), the age-stage-specific consumption (*C*_*xj*_), the age-specific net consumption rate (*q*_*x*_), the net consumption rate (*C*_0_), the cumulative consumption rate (*C*_*y*_) and the transformation rate (*Q*_*p*_)^31,47^.

### Molecular characterization

The DNA was extracted with a slight modification using the cetyl trimethylammonium bromide (CTAB) method^48^. In a sterile microcentrifuge, the female of *H. sedecimnotata* were grounded by adding 200 μL of preheated CTAB buffer. The samples were kept for 45 minutes at 65 °C. The tubes were centrifuged at 4 °C for 15 min at 16,000 g after adding an equal volume of chloroform: isoamyl alcohol mixture (24:1). A one-third volume of ice-cold isopropanol was added in the clear aqueous phase and the tubes were incubated at −20 °C overnight. The tubes were then centrifuged at 16,000 g at 4 °C for 20 minutes. It was dissolved in 20 μL of 1X TE buffer (pH 8.0) after washing the DNA pellet with 70% ethanol.

A set of COI primers [(F: C1-J-2183): CAA CAT TTT TTT TTT TTT GG and R (TL-2-N-3014-ANT): TGA AGT TTA AGT TCA ATG CAC] was used to amplify the DNA samples^49^. In a thermocycler (BIORAD DNA Engine, PTC-0200, Mexico) polymerase chain reactions (PCR) were performed. PCR reactions were conducted with a volume of 25.0 μL containing a buffer (10 mM Tris–HCl pH 9, 50 mM KCl), 200 µM dNTPs, 1.5 mM MgCl2, 10 pmol of both forward and reverse primers, 1 unit Taq DNA polymerase, and 4 ng of the extracted DNA. The PCR cycle included pre-denaturation at 94 °C for 2.0 min, 40 amplification cycles (denaturation at 94 °C for 1.0 min, annealing at 55.0 for 1.0 min, extension at 72 °C for 1.0 min, and final extension at 72 °C for 5.0 min. Following amplification, a 4 μL aliquot of PCR-amplified COI-primer products was separated from a 2% agarose gel containing 0.5 μg/mL ethidium bromide at 90 V for 45 minutes and viewed under UV light and data acquired from an image documentation system (Gel Doc XR System, Model: 1708170EDU, Bio-Rad Laboratories, Hercules, California, USA). A co-migrating 100 bp DNA ladder (MBI Fermentas) was compared to the size of the DNA fragment.

Sequences for *H. sedecimnotata* Indian isolate was deposited in the NCBI database and the accession number was MG720024. Homology searches conducted using the Basic Local Alignment Search Tool (BLAST) and differences in *H. sedecimnotata* COX-1 sequences were determined using the BioEdit version 7.0.5.3 sequence alignment editor.

The Clustal W program (a MEGA 4.0 sub-program) has been used to align the sequence and where necessary the alignment has been refined^50^. The statistics of molecular character, pairwise nucleotide sequence divergence, and base frequencies for each gene fragment were estimated using MEGA 4.0 software. Neighbor-Joining (NJ) and Minimum-Evolution (ME) for phylogenetic construction implemented in MEGA 4.0.

### Field evaluation of *Harmonia sedecimnotata* against *Aphis gossypii* on eggplant

This study was conducted on an experimental farm located in the ICAR Research Complex for NEH Region, Mizoram Centre, Kolasib, Mizoram, India at an altitude of 650 m, 24.12°N latitude and 92.40°E longitude and has a mild-tropical agroclimate. For the seasons 2013–2014 (season 1) and 2014–2015 (season 2), field experiments were conducted in eggplant (var. Pusa Purple Round). Treatment of 5 replicates arranged in a randomized complete block design (RCBD) has been applied. During two seasons, average daily temperatures in the open field ranged from 20.8 to 32.3 °C with relative humidity ranging from 74 to 85.0%; low rainfall during the observation period.

The predator’s two releases were made at 30 days apart. Each release was released at five rates of 10, 20, 30, 40, and 50 adults (1 F: 1 M) per plot (100 m^2^). The first release was made during the vegetative stage when the aphid population was above the economic threshold (10–29% affected plants). The second release covered new leaves and shoots and increased populations in newly emerging aphids nymphs and adults, making it 30 days after the first release. In the same plants/plots, both releases were made. During the study, the control was free to release coccinellids. In order to avoid the movement of predators from one treatment to another, each treatment was separated by a distance of 25 m and no insecticide spray was given throughout the experimental period.

Nymph and adult aphid densities were estimated 24 h before each release, and observations of post-treatment were at intervals of fortnight. Densities of melon aphids were determined by counting individuals on the upper and under sides of three leaves from the bottom, middle, and top. Thirty leaf samples (each “leaf sample” consisted of three leaves from bottom, middle, and top) by selecting ten plants from each replication. The plants were randomly examined on each sample date for nymph and adult stages of melon aphids in each plot. Mean aphid population per leaf was estimated by an average of three leaves (bottom, middle and top). The aphid population was recorded with the aid of a magnifying glass (MAG, Generic Brand, India). Data were estimated weekly in the canopy area between 8.0 and 10.0 h. For each release, the percentage reduction in aphids over control was calculated.

### Statistical analysis

Data were analyzed using SAS Software Version 9.3^51^ for biology, and consumption rate with completely randomized design (CRD) and field assessment with RCBD. Data on total, per-day, and percentage consumption for larvae or adults were analyzed using analysis of variance (ANOVA) and the ANOVA was performed on the original values and the means were analyzed by least squares means (LSD) and the mean separated using Tukey’s HSD test at *P* ≤ 0.001. Field assessment data were transformed into square root and arc sine wherever required, as described by Poisson for statistical analysis^52^. ANOVA was done on the transformed values of field evaluation data and the same values were analyzed by least squares means (LSD) and separated using Tukey’s HSD test at *P* ≤ 0.001.
